# Supplementary material for: ZWA: Viral genome assembly and characterization hindrances from virus-host chimeric reads; a refining approach
Source: PLoS Comput Biol. 2021 Aug 9;17(8):e1009304. doi: 10.1371/journal.pcbi.1009304 (PMC8376068; doi:10.1371/journal.pcbi.1009304)
Supplement: S1 Table — (DOCX) [file pcbi.1009304.s001.docx]

S1 Table. GenBank Accession IDs of virus sequences containing ribosomal sub-sequences along with the starting position and length of the moieties.

| **ACCESSION ID** | **STARTING POSITION** | **LENGTH** |
| --- | --- | --- |
| *MN053791.1* | 3093 | 49 |
| *MK100570.1* | 2856 | 52 |
| *MK100569.1* | 4009 | 55 |
| *JA417780.1* | 1 | 61 |
| *NC_038286.1* | 1 | 61 |
| *AY034063.1* | 275 | 62 |
| *MG770349.1* | 1 | 63 |
| *MH052023.1* | 1010 | 68 |
| *MN053803.1* | 3045 | 74 |
| *MN053801.1* | 3100 | 74 |
| *KF892040.1* | 6391 | 76 |
| *KU178986.1* | 1 | 79 |
| *KJ716849.1* | 4427 | 80 |
| *NC_008168.1* | 25903 | 89 |
| *DQ333351.1* | 25903 | 89 |
| *NC_018464.1* | 838 | 90 |
| *HE795107.1* | 838 | 90 |
| *AB972431.1* | 1 | 95 |
| *KP642119.1* | 1 | 97 |
| *MH892403.1* | 15565 | 110 |
| *MN033932.1* | 2725 | 114 |
| *KM972720.1* | 1 | 116 |
| *EU981569.1* | 132 | 126 |
| *JX291540.1* | 1792 | 131 |
| *HQ442266.1* | 374 | 144 |
| *MF289414.1* | 12143 | 160 |
| *KC786228.1* | 34 | 160 |
| *KF478765.1* | 1 | 178 |
| *JX185662.1* | 415 | 191 |
| *JX185663.1* | 507 | 194 |
| *JX185666.1* | 414 | 194 |
| *JX185667.1* | 421 | 194 |
| *MK332105.2* | 10238 | 204 |
| *LN680393.2* | 1577 | 390 |
| *KU746280.1* | 1 | 582 |
| *MF094128.1* | 1 | 623 |
| *KU746283.1* | 1 | 1024 |
| *MH883318.1* | 134427 | 5560 |
