# Supplementary material for: ZWA: Viral genome assembly and characterization hindrances from virus-host chimeric reads; a refining approach
Source: PLoS Comput Biol. 2021 Aug 9;17(8):e1009304. doi: 10.1371/journal.pcbi.1009304 (PMC8376068; doi:10.1371/journal.pcbi.1009304)
Supplement: S1 Fig — X-axis shows the bp positions of the reference and Y-axis shows how many reads of the mock file were aligned on each position. (DOCX) [file pcbi.1009304.s002.docx]

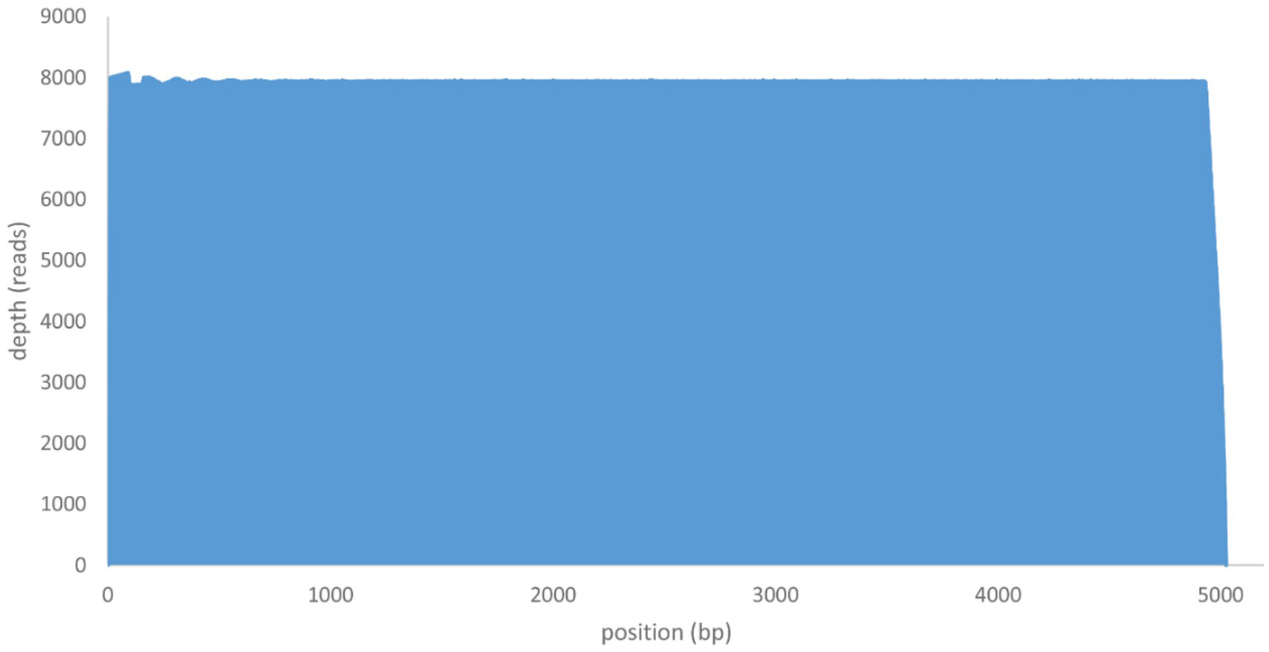


S1 Fig. Alignment of mock human 28S rRNA reads on the human 28S reference sequence (M11167.1) for validation. X-axis shows the bp positions of the reference and Y-axis shows how many reads of the mock file were aligned on each position.
