# Supplementary material for: ZWA: Viral genome assembly and characterization hindrances from virus-host chimeric reads; a refining approach
Source: PLoS Comput Biol. 2021 Aug 9;17(8):e1009304. doi: 10.1371/journal.pcbi.1009304 (PMC8376068; doi:10.1371/journal.pcbi.1009304)
Supplement: Attachment — Submitted filename: Comments.docx [file pcbi.1009304.s003.docx]

Comments to Authors:

In this manuscript, the authors investigated the effect of chimeric reads on the viral genome assembly first and then proposed a pipeline to solve this problem.

The study was well designed, but authors should also elucidate some details clearly before the manuscript was accepted.

Major Comments:

1. The main idea of processing the chimeric reads is to remove the overlapped part of host rRNAs and use the remaining reads for assembly. However, this idea may be not novel. I searched another paper with the PMID 27605096 [1]. In this paper, they also described a pipeline in Figure 1 and it seems the same with ZWA. So, the novelty of this manuscript is suspected.

2. In Figure 3, you also found a significant portion of rRNA located in the middle of the viral sequences. So, how did you handle this type of reads?

3. What’s your standard for the excision of hybrid reads? How long will you keep the excised reads with?

4. Did you compare you pipeline with others? What’s the performance of your pipeline? Running time? Accuracy? ROC?

5. In Figure 7B, there are still 151 reads that were not cured and what are they? So, are the 99 reads in Figure 7C the same? How many times of iteration does ZWA need?

Minor Comments:

1. For Figure 6, please describe the subfigure separately.

[1] Berthet N, Descorps-Declère S, Nkili-Meyong AA, Nakouné E, Gessain A, Manuguerra JC, Kazanji M. Improved assembly procedure of viral RNA genomes amplified with Phi29 polymerase from new generation sequencing data. Biol Res. 2016 Sep 7;49(1):39. doi: 10.1186/s40659-016-0099-y. PMID: 27605096; PMCID: PMC5015205.
