# Supplementary material for: ZWA: Viral genome assembly and characterization hindrances from virus-host chimeric reads; a refining approach
Source: PLoS Comput Biol. 2021 Aug 9;17(8):e1009304. doi: 10.1371/journal.pcbi.1009304 (PMC8376068; doi:10.1371/journal.pcbi.1009304)
Supplement: Attachment — Submitted filename: ANSWERS to reviewers.docx [file pcbi.1009304.s004.docx]

We would like to thank the reviewers for their time and their invaluable input in improving our manuscript.

Please find below our responses to the reviewers’ comments. The manuscript has also been amended to reflect these improvements (related passages are highlighted and line numbers have been added to assist the reviewing process)

**Reviewer #1:**

The paper presents an improved approach for the viral genome assembly using virus-host chimeric reads but reducing artifacts.

***Comment:***
 First of all, I advise to improve the title to a new one: "METHOD-NAME: a refined (?) viral genome assembly approach by characterization hindrances from virus-host chimeric reads".

***Answer:***
 We thank the reviewer for this suggestion and have amended our title to more suitably reflect this work (Line 1)

***Comment:***
Next, I have a few major comments to be addressed:
1. The authors presented an evidence that a group of deposited sequences encompassed ribosomal RNA (rRNA) contamination in viral genomes after genome assembly. I would suggest to check not only ribosomal RNA, but whole human genome sequences, since the contamination may be done by the pieces of "junk DNA" and/or coding regions from human genome as well as other hosts. Please provide an analyzes per host and per all possible viruses by a categories: DNA viruses and RNA viruses. For the viruses' classification purposes the recent publication in the FEBS journal may be used: <https://doi.org/10.1111/febs.15835>

***Answer:***
 Once again, thank you for this suggestion. We have expanded our search using the whole human transcriptome reference sequence (GRCh38) identifying multiple viral genomes contaminated with host sequences. However, we haven’t managed to solve computationally the decontamination procedure that should take into account that the whole human transcriptome is 60.000 times bigger than the ribosomal RNA we used here, making the presented pipeline unable to handle the magnitude of information in a timely manner. For this reason, a more computationally advanced version of the algorithm is required which we are currently investigating.
 However, as we mentioned in the manuscript most novel viruses from new species are discovered with total RNAseq which is comprised by 85% ribosomal RNA and thus it is anticipated that the majority of chimeras encompass ribosomal moieties. We should stress here, as stated in the manuscript, that wetlab ribodepletion is very species specific and the current kits focus on dominant model organisms.
 If such an improvement in the manuscript is a total requirement we could accelerate the development of a more time-efficient pipeline possibly with the use of super- or cloud- computing. Afterall, the algorithm presented here can be scaled beyond the currently used tools we have implemented here which carry their own time efficiency restrictions and were used as proof-of-concept.

***Comment:***
2. Now the question arises: how do you distinguish between the "contamination" by rRNA and the horizontal gene transfer (HGT) events? Please collected reported HGT cases, make a test set, and verify your method such that you will be able to separate between the artifacts and the real HGT events. Please make available the HGT cases for users as well as other test sets.

***Answer:***
 Thank you for this question. Screening the literature for naturally occurring recombinants between viral genomes and host sequences we identified 2 paradigms where ribosomal RNA sequences were naturally incorporated. These 2 previous reports date back to 1989 and 1994 and presented a presumable incorporation of ribosomal RNA (28S) in influenza and poliovirus respectively. Since then, there has been no other similar report. However, we must stress that in these early days of molecular diagnosis, PCR artifacts were not recognised. Furthermore, as these 2 genomes are not included in GenBank our search algorithm did not identify them.
 After the introduction of NGS for genome characterisation, multiple reads (after deduplication) are required to support rare genome rearrangement events. Real recombinants would be expected to have a higher read representation on the recombination site. In our experimental dataset such chimeric reads were singletons or duplicates of the same read (PCR duplicates). The manuscript has been amended to include this comment (Lines 247-248)
 The aforementioned paradigms are represented in the table below and this comment is reflected in our amended discussion. (Lines 360-368)

| **Publication** | **Year of publication** | **No. of nucleotides** | **Non-viral sequence type** | **Virus** |
| --- | --- | --- | --- | --- |
| Khatchikian et al. PMID: 2544809 | 1989 | 54nt | 28S ribosomal | Influenza |
| Charini et al. PMID: 8083991 | 1994 | 15nt | 28S ribosomal | Poliovirus |

***Comment:***
3. Please show the statistics on the observed chimeric cases for different hosts, not just for the human host, but also for all the hosts/viruses collected by the categories mentioned in 1. The "goal standard contigs" set should be produced such that any viral analyses may trim easily these previously observed chimeric reads in human/non-human hosts.

***Answer:***
 Thank you for pointing this out. Figure 2 legend has been amended to stipulate that the Venn diagram includes all host species.
 We have also created a table with all the reported chimeric sequences in GenBank along with the contamination length and starting position which will is now provided as a supplementary table. (SUPPLEMENTARY TABLE 1 and Lines 234-236 of the manuscript)

| **ACC. ID** | **START POS** | **LENGTH** |
| --- | --- | --- |
| MH883318.1 | 134427 | 5560 |
| KU746283.1 | 1 | 1024 |
| MF094128.1 | 1 | 623 |
| KU746280.1 | 1 | 582 |
| LN680393.2 | 1577 | 390 |
| MK332105.2 | 10238 | 204 |
| JX185663.1 | 507 | 194 |
| JX185666.1 | 414 | 194 |
| JX185667.1 | 421 | 194 |
| JX185662.1 | 415 | 191 |
| KF478765.1 | 1 | 178 |
| KC786228.1 | 34 | 160 |
| MF289414.1 | 12143 | 160 |
| HQ442266.1 | 374 | 144 |
| JX291540.1 | 1792 | 131 |
| EU981569.1 | 132 | 126 |
| KM972720.1 | 1 | 116 |
| MN033932.1 | 2725 | 114 |
| MH892403.1 | 15565 | 110 |
| KP642119.1 | 1 | 97 |
| AB972431.1 | 1 | 95 |
| HE795107.1 | 838 | 90 |
| NC_018464.1 | 838 | 90 |
| DQ333351.1 | 25903 | 89 |
| NC_008168.1 | 25903 | 89 |
| KJ716849.1 | 4427 | 80 |
| KU178986.1 | 1 | 79 |
| KF892040.1 | 6391 | 76 |
| MN053801.1 | 3100 | 74 |
| MN053803.1 | 3045 | 74 |
| MH052023.1 | 1010 | 68 |
| MG770349.1 | 1 | 63 |
| AY034063.1 | 275 | 62 |
| JA417780.1 | 1 | 61 |
| NC_038286.1 | 1 | 61 |
| MK100569.1 | 4009 | 55 |
| MK100570.1 | 2856 | 52 |
| MN053791.1 | 3093 | 49 |

**Reviewer #2:**

***Comment:***

In this manuscript, the authors investigated the effect of chimeric reads on the viral genome assembly first and then proposed a pipeline to solve this problem.

The study was well designed, but authors should also elucidate some details clearly before the manuscript was accepted.

***Answer:***
 We thank the reviewer for the encouraging words. Please find below our answers point by point to your concerns.

***Comment:***

1. The main idea of processing the chimeric reads is to remove the overlapped part of host rRNAs and use the remaining reads for assembly. However, this idea may be not novel. I searched another paper with the PMID 27605096 [1]. In this paper, they also described a pipeline in Figure 1 and it seems the same with ZWA. So, the novelty of this manuscript is suspected.

***Answer:***
 We would like to thank the reviewer for highlighting this article as such methodologies are the reason we developed ZWA algorithm. There are fundamental differences between Berthet et al. algorithm and our approach. We present an excerpt of their methodology section here marking with A, B and C the differentiating parts and addressing them separately*.*

*“The quality of the reads was initially assessed by FastQC. The mouse genome sequence was filtered by mapping the selected reads on the Mus musculus Mn10 sequence using Bowtie 2.0 software with the “very sensitive” flag option [7]. (A) All remaining reads corresponding to viral sequences were obtained based on “similarity-based” approach and used BLASTN and BLASTX with a defined number of targeted sequences available in sequence databanks (L22089, DQ294633.1 and KF680222.1) (B). All viral reads were selected according to the percentage of identity (a minimum of 75 %) between the reads and reference sequences and a minimum alignment length of 60 bases including indel. In order to improve the assemblage quality of viral genomes, only the region of each read matching BLAST results was selected and kept (Fig. 1). This way, all non-viral sequences potentially associated with a viral sequence inside the same read generated during the retrotranscription step were removed. The selected reads were assembled with different software, such as ABySS, Ray and SPAdes (version 3.0; 3.5 and 3.6) with different k values used to build the Bruijn graph [8, 9]. (C) All genome assemblies were evaluated using the QUAST tool such as the number of obtained contigs, the size of the largest contig, the N50 and L50 and finally, the coverage of the genome obtained [10]. The proportion of reads which unmapped on generated contig(s) for each set of data was determined by mapping, by using Bowtie 2.0 software with the “very sensitive” flag option and “End to End” as the alignment type in the Geneious R9 software. All chimeric reads were identified from a tabular output of a BLAST generated file which contained matching positions from reads against BLAST hits. A read was considered to be chimeric if its entire sequence did not belong to the alignment. (D)”*

A. The presented algorithm is using the mouse genome reference sequence to discard reads which contain host sequences using the preset [*--very-sensitive*](http://bowtie-bio.sourceforge.net/bowtie2/manual.shtml#bowtie2-options-very-sensitive) that is the same as running with options: *-D 20 -R 3 -N 0 -L 20 -i S,1,0.50*. These most likely contain a significant portion of the chimeric reads which ZWA is designed to clean and utilise. The rest of chimeric reads are then fed to step B. Thus, we believe that the presented algorithm may discard important virus information for a successful assembly.
B. The authors here are using a similarity-based approach against 3 known virus reference sequences. Our approach is virus-agnostic and works without prior knowledge of the viruses in the samples. The ZWA algorithm is a universal method that may easily be applied in viral metagenomics. Berthet et al. approach requires a database in order to perform in viral metagenomics.
C. The assembly that followed the similarity-based approach on the reference viral genome, although technically *de novo,* by using a reference based preselection of reads renders it as guided in contrast to our approach that is entirely *de novo* (no virus reference or virus database needed)
D. This is another point that the authors emphasize that chimeric reads were identified according to BLAST alignment against virus reference sequences.

We have included the reviewer’s indicated citation with a respective argument in the discussion section (Lines 353-355)

***Comment:***
2. In Figure 3, you also found a significant portion of rRNA located in the middle of the viral sequences. So, how did you handle this type of reads?

***Answer:***	Thank you for this question. Figure 3 only refers to assembled viral sequences found in NCBI Nucleotide and not sequences in our paradigm but those indeed exist. In our experimental dataset multi-chimeric reads were processed by repeated runs of the pipeline trimming from each end (3΄, 5΄) every time. Unfortunately, very complicated multi-chimeric reads may result in some very small loss of viral sequences as depicted in Figure 7.

***Comment:***
3. What’s your standard for the excision of hybrid reads? How long will you keep the excised reads with?

***Answer:***
 As depicted in our code we pinpoint the hybrid part’s orientation using the starting and end positions of the rRNA:

if (( $(echo "${hybrid_start} > ${percentage5} && ${hybrid_end} < ${percentage95}" |bc -l) ));
then
echo ${dirty_seq} >> cleaninglist.txt
continue
fi

If the majority of the sequence is ribosomal with only 5% non-ribosomal on each extremity (5΄ or 3΄) the algorithm discards the read. This can be considered as a lost read.

if (( $(echo "${hybrid_end} > ${percentage90}" |bc -l) ));
then
echo ">"${dirty_seq} >>clean_seqs.fasta
echo ${target_seq:0:${hybrid_start}} >>clean_seqs.fasta
echo ${dirty_seq} >> cleaninglist.txt
continue
fi

If the hybrid part exceeds the 90^th^ percentile of the read the algorithm keeps all nucleotides starting from 5΄ until the start of the hybrid part.

if (( $(echo "${hybrid_start} < ${percentage10}" |bc -l) ));
then
echo ">"${dirty_seq} >>clean_seqs.fasta
echo ${target_seq:${hybrid_end}} >>clean_seqs.fasta
echo ${dirty_seq} >> cleaninglist.txt
continue
fi

If the hybrid part starts inside the 10^th^ percentile of the read the algorithm keeps all nucleotides starting from the hybrid end until the 3΄.

The trimmed reads are considered clean and are pooled together with the pure non-ribosomal reads for the assembly procedure.

***Comment:***
4. Did you compare you pipeline with others? What’s the performance of your pipeline? Running time? Accuracy? ROC?

***Answer:***	The other known pipelines discard ribosomal reads along with the chimeric ones and thus it’s not possible to compare them with our own pipeline. In the manuscript we are comparing our results with SORTMERNA which also discards reads, as mentioned. All pipelines that discard ribosomal reads have a shorter running time but lose out on information.
 Accuracy and ROC are metrics which validate predictive values of procedures and cannot be applied to this kind of pipeline. To validate the accuracy of ZWA as per the reviewers request, we created a synthetic dataset which contained human ribosomal random reads and 20% chimeric reads. Testing ZWA on that dataset yielded 100% accuracy in identifying the chimeric reads. 20% of the produced cleaned virus sequences lost their first nucleotide. This is, possibly, an artifact as there is a chance that the first nucleotide of the non-ribosomal sequence may align to the ribosomal sequence (the theoretical probability for any random nucleotide is 25%).

***Comment:***
5. In Figure 7B, there are still 151 reads that were not cured and what are they? So, are the 99 reads in Figure 7C the same? How many times of iteration does ZWA need?

***Answer:***	After the first round of chimeric read trimming, chimeric reads were reduced to 151 from 807. These 151 reads are still chimeric reads and the result of trimming original multi-chimeric reads. In the second round of trimming 99 reads are still chimeric and are possibly the remnants of very complicated original chimeric reads. There is a possibility that in a third round of trimming some of them would be cleaned. However, the final gain would be small while wasting time with multiple rounds of trimming. Thus, we believe that two runs clean the vast majority of chimeric reads while discarding possibly very complicated multi-chimeric reads.

***Comment:***
1. For Figure 6, please describe the subfigure separately.

***Answer:***
 Thank you for pointing this out. Figure 6 legend has been corrected to reflect the subfigures.
