# Supplementary material for: ZWA: Viral genome assembly and characterization hindrances from virus-host chimeric reads; a refining approach
Source: PLoS Comput Biol. 2021 Aug 9;17(8):e1009304. doi: 10.1371/journal.pcbi.1009304 (PMC8376068; doi:10.1371/journal.pcbi.1009304)
Supplement: Attachment — Submitted filename: answers.docx [file pcbi.1009304.s005.docx]

Once again, we would like to thank the reviewers for their efforts, providing critical input in making this manuscript suitable for publication.

Please find below our responses to the reviewers’ comments. The manuscript has also been amended to reflect these improvements (related passages are highlighted and line numbers have been added to assist the reviewing process). This amended manuscript also includes suggestions from the editorial office (eg. the addition of the author summary)

**Reviewer #1:**

***Comment:***
The manuscript has been improved significantly as a result of the revision.

***Answer:***
We share the same view as the reviewer on this. Their feedback was significant and has made the manuscript a better reading experience. Truly thank you for this.

***Comment:***
I suggest the following technical improvement:

please develop a more time-efficient pipeline possibly with the use of GPU- or cloud-computing to extend the presented proof-of-concept algorithm and provide the online availability for the scientific community as a link/availability issue in the manuscript.

***Answer:***
Thank you for the suggestion. Due to the nature of the pipeline being based on a bash script a GPU-based approach is not possible. Furthermore, the tools implemented in the pipeline are extensively tested by the community and recommended (such as Blast+, BWA, samtools and Trinity) but they also bring their own complexity to the algorithm. Fortunately, the algorithm itself allows for scaling via implementation using a different approach, which we will explore in the future. To ameliorate the hardware burden of the current pipeline we have created a ready to deploy Docker image which can provide ease of use for the readers and the ability for ZWA to be deployed on any cloud infrastructure, taking advantage of more parallel threads, as advised by the reviewer.

The manuscript has been amended to include this publicly available Docker image <https://hub.docker.com/repository/docker/ndovroli/zwa/general> (Lines 226-229). The availability of the Docker image is also described on Github.

**Reviewer #2:**

***Comment:***
Authors have responded to all the comments and modified the manuscript. I would suggest acceptance of the manuscript with the title ZWA: Viral genome assembly and characterization hindrances from virus-host chimeric reads; a refining approach.

***Answer:***
Thank you for making this possible. We appreciate all your comments and suggestions in this process.
